# Deep brain stimulation programming for intractable obsessive–compulsive disorder using a long pulse width

**DOI:** 10.3389/fpsyt.2023.1142677

**Published:** 2023-06-29

**Authors:** Emily Beydler, Lauren Katzell, Kevin Putinta, Richard Holbert, Brent Carr

**Affiliations:** ^1^College of Medicine, University of Florida, Gainesville, FL, United States; ^2^Department of Psychiatry, University of South Alabama, Mobile, AL, United States; ^3^Department of Psychiatry, University of Florida, Gainesville, FL, United States

**Keywords:** deep brain stimulation, intractable OCD, psychiatric deep brain stimulation, anterior limb of the internal capsule, monopolar settings, case reports deep brain stimulation, case reports

## Abstract

**Introduction:**

Around 25% of patients with obsessive–compulsive disorder (OCD) do not respond to medication or psychotherapy, producing significant impairment and treatment challenges. Deep Brain Stimulation (DBS) has been shown in multiple blinded trials to be a safe and durable emerging option for treatment-refractory OCD. Intraoperative device interrogation offers a theoretical anchor for starting outpatient DBS programming; however, no definitive post-operative programming algorithm for psychiatrists exists currently.

**Case:**

Here we present a 58-year-old female with childhood-onset, severe, intractable OCD with multiple failed trials of psychotherapy, medication, and electroconvulsive therapy. After interdisciplinary evaluation, she underwent bilateral electrode implantation targeting the anterior limb of the internal capsule, nucleus accumbens (ALIC/NAc). Intraoperative interrogation afforded sparse information about a preferred lead contact or current density target. Subsequent outpatient interrogation consisted of systematic and independent mapping using monopolar cathodic stimulation with constant current. Modulating bipolar and triple monopolar configurations, amplitude, and pulse width all failed to induce observable effects. Given negligible interrogation feedback, we created an electrical field through the ALIC bilaterally, using the three most ventral contacts to create triple monopoles, with a long pulse width and moderate amperage.

**Conclusion:**

Three months post-programming, the patient reported significant improvement in OCD symptoms, particularly checking behaviors, with response sustained over the next several months. As with our case, the majority of DBS lead contacts do not induce affective or physiological markers in patients, complicating programming optimization. Here, we discuss an approach to titrating various stimulation parameters and purported mechanisms of physiological markers in DBS for OCD.

## Introduction

Structural and functional neural circuit mapping of OCD is some of the most robust in the psychiatric literature, with strong evidence for aberrant hyperconnectivity in cortico–striato–thalamo–cortical (CSTC) circuitry, known as the frontostriatal model (1, 2). Recent research has also shown the involvement of other neural networks at interplay with the CSTC circuit (3, 4). Despite the strength of this work, treatment for OCD remains suboptimal, with upwards of one-third of patients not fully responding to psychotherapy and psychopharmacology (5, 6). Furthermore, intractable OCD, in which multiple treatment regimens have failed, affects up to 10% of patients (7). Historically, partial response for such cases was obtained through the use of psychosurgical capsulotomy, in which the anterior limb of the internal capsule (ALIC) was ablated to disrupt frontostriatal circuits during the 1940s and 1950s (8, 9). The initial rationale behind Deep Brain Stimulation (DBS) was to create a reversible lesion similar to a capsulotomy (10, 11).

For bilateral ventral caudate/ventral striatum (VC/*VS*) DBS lead placement, the ventromedial region of the nucleus accumbens (NAc) is stimulated through the most ventral contact, with the middle two contacts stimulating the ventral portion of the ALIC. This configuration results in similar targets to a ventral capsulotomy, though the VC/*VS* target has moved posteriorly over time due to refinement in targeting methods, resulting in better clinical outcomes (9, 12). Results from a double-blind controlled trial of DBS for OCD showed comparable efficacy to psychosurgery, greater response rates, and decreased symptom severity as a function of more recent posterior lead placement versus its antecedent DBS targets (9, 13).

In addition to its clinical promise, DBS is likely to be cost-effective over long-time periods, especially when a rechargeable internal pulse generator (IPG) is used (14). However, overall access to DBS remains an issue for many patients with intractable OCD like ours due to inconsistent coverage by insurers, who frequently incorrectly cite the treatment as experimental (11, 15). DBS for OCD is evidence-based treatment under a humanitarian device exemption (HDE) status by the FDA, versus experimental treatments which fall under an investigational device exemption (IDE) (11, 15). There is currently a call by leaders in the field to expand access to this treatment, both by increasing the number of DBS-trained psychiatrists comfortable with the management of these patients and through expanded insurance access (11). Of note, the lack of insurance coverage for DBS for OCD violates 2008 mental-health parity laws, which require equal coverage for medical and mental health conditions by major insurers, and notably, DBS is covered by major insurers for neurological conditions like dystonia, which also falls under an HDE (15). This case report describes a method we used to evaluate and adjust different stimulation parameters. We also examined their potential mechanisms of physiological side effect and response to stimulation and how these related to her clinical outcomes.

### Case

#### Patient information & clinical findings

Our patient is a 50 y.o. female who presented for neurosurgical evaluation for a 35-year history of intractable OCD. Her symptoms began following a childhood trauma, and her obsessions centered on safety. She described intrusive thoughts of harming others, checking rituals, and feelings of incompleteness. She no longer could drive or leave her house alone due to these symptoms. She worked in a complex healthcare occupation previously but left her job due to excessive time spent in documentation due to OCD symptoms. Previous treatment trials included psychotherapy, various psychopharmacological trials (including SSRIs, atypical antidepressants, atypical antipsychotics, and benzodiazepines), and electroconvulsive therapy.

#### Diagnostic assessment

This patient was evaluated at the University of Florida Center for Movement Disorders and Neurorestoration as a collaboration of the departments of Psychiatry, Neurology, and Neurosurgery. Prior to recommending surgery, a risk versus benefit analysis was performed by a multidisciplinary team (psychiatrist, psychologist, neurologist, and functional neurosurgeon), which reviewed all past treatments, procedures, and evaluations to ensure the appropriateness of the candidate. Psychiatric diagnoses were based on a review of medical records and clinical interviews.The patient met DSM-V criteria for OCD with an Obsessive Compulsive Scale (Y-BOCS), with a history of treatment-refractory OCD symptoms since age 15 causing suffering and functional impairment. Her symptomatology was supported by a Y-BOCS of 36 at the time of the initial DBS consultation, indicating extreme severity (scores 32–40). Previous psychotherapy, pharmacological, and electroconvulsive therapy trials were deemed adequate, and she was deemed a potential candidate for deep brain stimulation therapy to treat her debilitating, medication-refractory OCD symptoms.

While she was deemed a surgical candidate at this time, the next 7 years were spent on medication optimization because her insurance would not cover the procedure. Upon changing insurance, the patient was again deemed a surgical candidate after repeat evaluation by a multidisciplinary team. She underwent successful bilateral VC/*VS* Medtronic device implantation and initial device programming occurred over a 3-month period. Her medication regimen was fluvoxamine 100 mg 3 times per day, olanzapine 10 mg at night, and clonazepam 1 mg 3 times per day, and remained unchanged over the following year throughout DBS implantation and optimization.

#### DBS implantation

**Figure 1:**
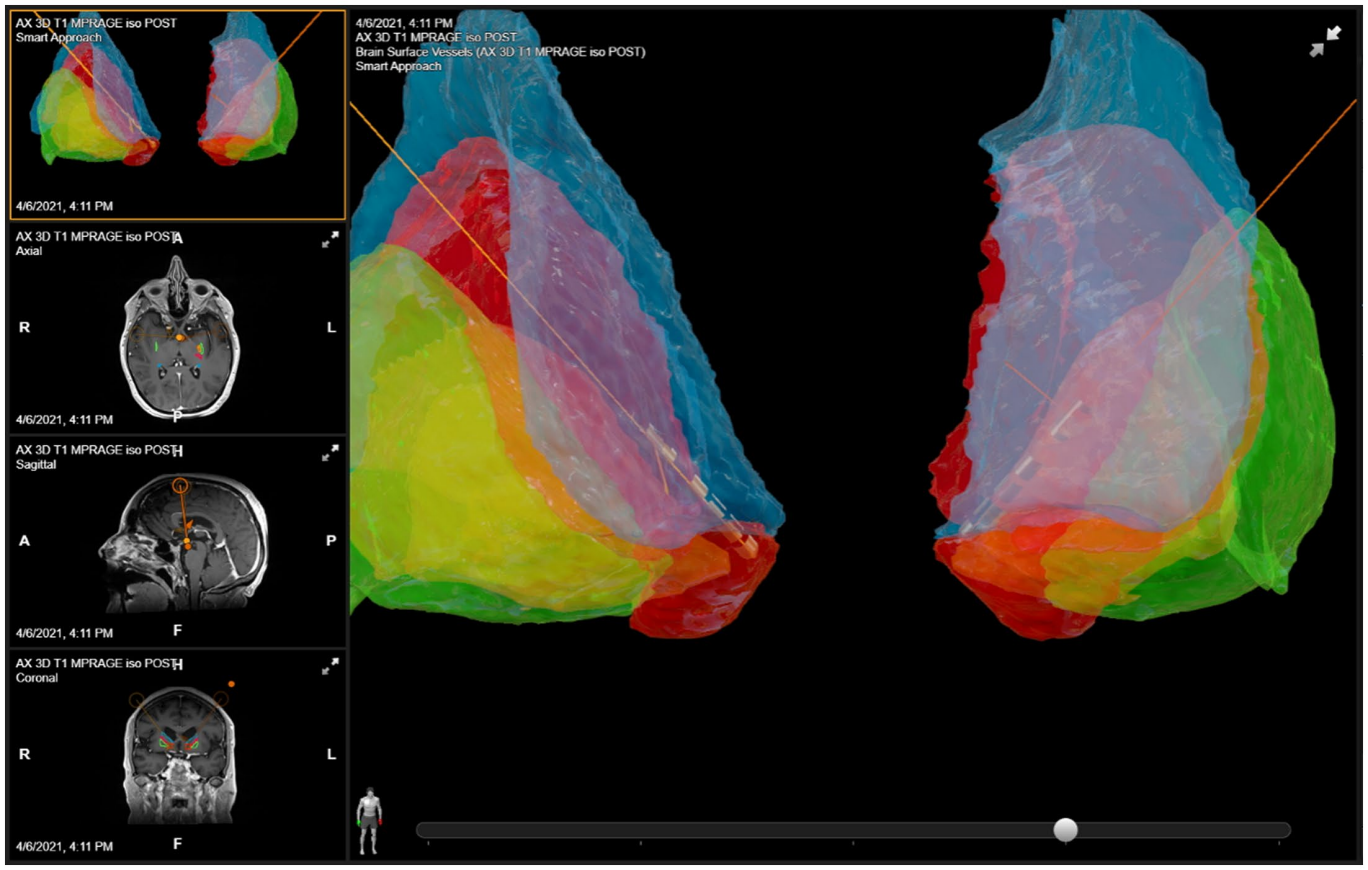
DBS lead placement. Images were generated using patient-specific anatomical mapping algorithm lead localization software in *BrainLAB Elements* (Pink: internal capsule, Orange: GPi, Green: Putamen, Blue: Caudate).

**Figure 2:**
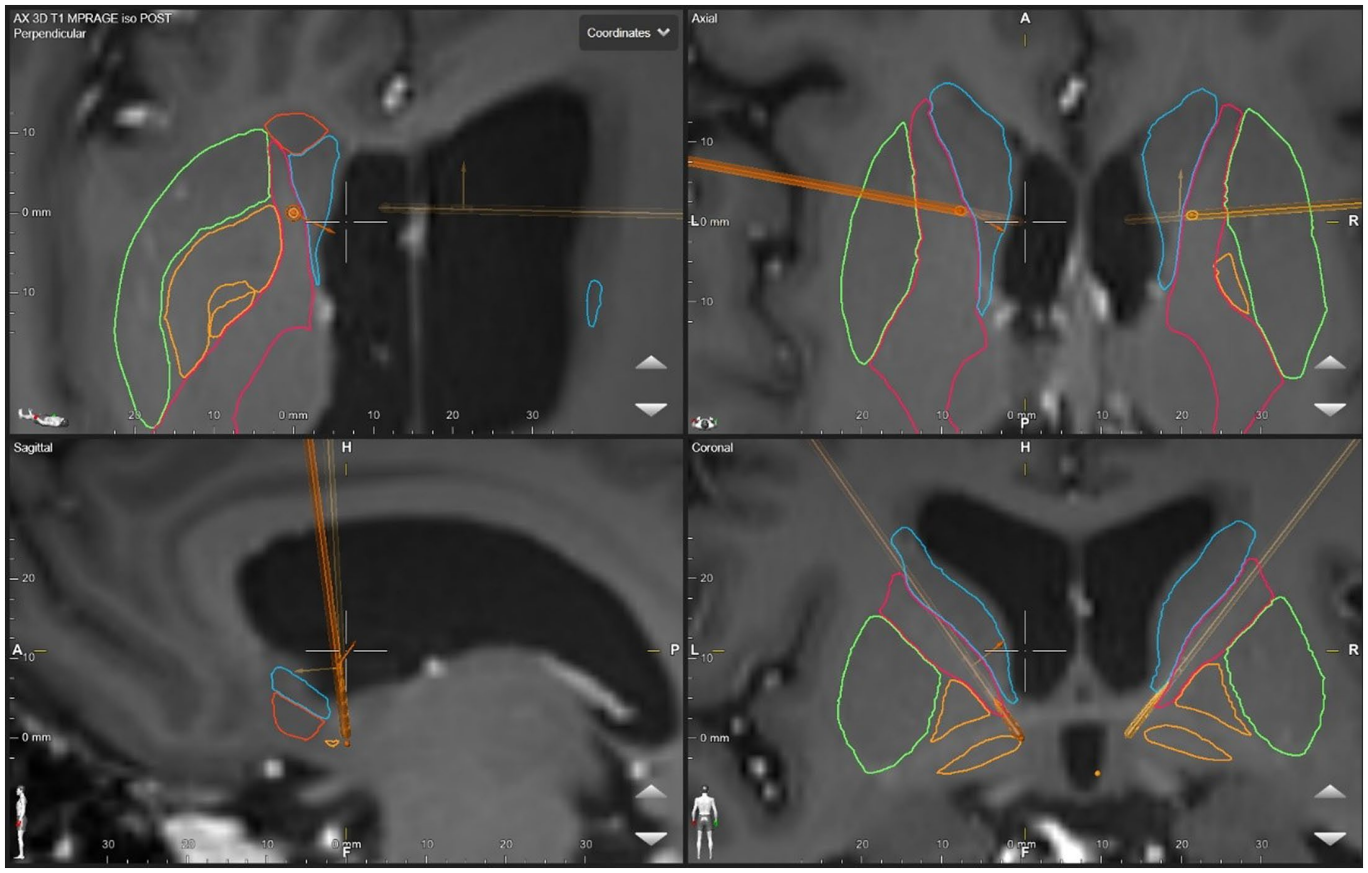
DBS lead placement. Images were generated using patient-specific anatomical mapping algorithm lead localization software in *BrainLAB Elements* (Pink: internal capsule, Orange: GPi, Green: Putamen, Blue: Caudate).

**Figure 3:**
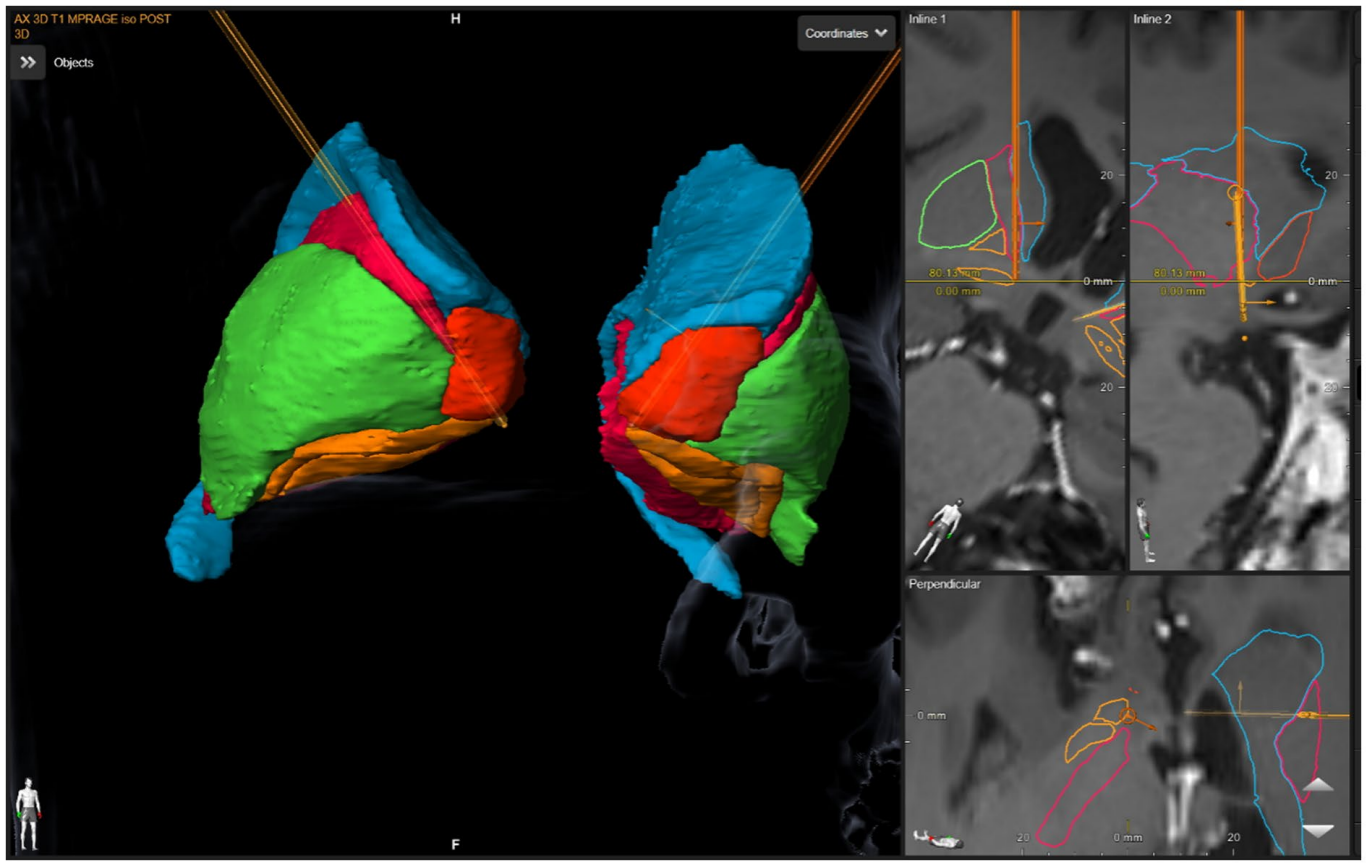
DBS lead placement. Images were generated using patient-specific anatomical mapping algorithm lead localization software in *BrainLAB Elements* (Pink: internal capsule, Orange: GPi, Green: Putamen, Blue: Caudate).

The anatomical target was the same for both the left and right sides. The DBS targeting utilized a stereotactic CT scan fused with MRI and morphed to a deformable atlas and was deemed successful. Although coordinates are less meaningful in such individual cases due to high patient-to-patient variability, we have included a patient-specific figure showing the lead position (Figures 1–3). Additionally, it should be noted that variability in DBS settings across leads may be explained by the variability of lead implantation within the targeted brain area. After implantation, there was no difference in the positions of the left and right leads.

#### Therapeutic interventions

At 2 months post-implantation, we attempted an initial systematic, exploratory, trial-and-error approach, assessing the effects of various stimulation parameters predominately through variation in amperage amplitude across various contacts. Frequency was held constant throughout at 135 Hz. Although monopolar stimulation lacks precision, it allows for a larger electrical field spread across the targeting region. For our purposes this broader, less precise, field would be of benefit for exploring regions that might elicit an acute positive emotional or affective response such as feelings of euphoria, mirth, or smile.

Monopolar interrogation consisted of testing all eight contacts (4 each, bilaterally) individually and systematically at 135 us, 190 us, 210 us, and 260 us. For each given pulse width, amperage was titrated upwards in 0.5 mA increments every 60 s. At longer pulse widths of 210us and 260us, she reported side effects– feeling “clammy and sweaty” and nauseated at the most ventral, right contact at 4.0 mA. We noticed no affective stimulus response elicited by the DBS stimulation.

Double monopolar settings were interrogated, and we used a similar titration pattern, pairing the two most ventral contacts, then pairing the middle contacts, holding the frequency at 135 Hz again. No stimulus effects, i.e., no mirth or smile were noted with any settings. Consistent with monopolar interrogation, the most ventral pair on the right induced side effects of “clamminess” and feeling “hot” at 3.3 mA. And for the left ventral most contact, “hot flashes” and “nausea” occurred at 3.5 mA. No euphoria, smile nor mirth was noted.

**Figure 4:**
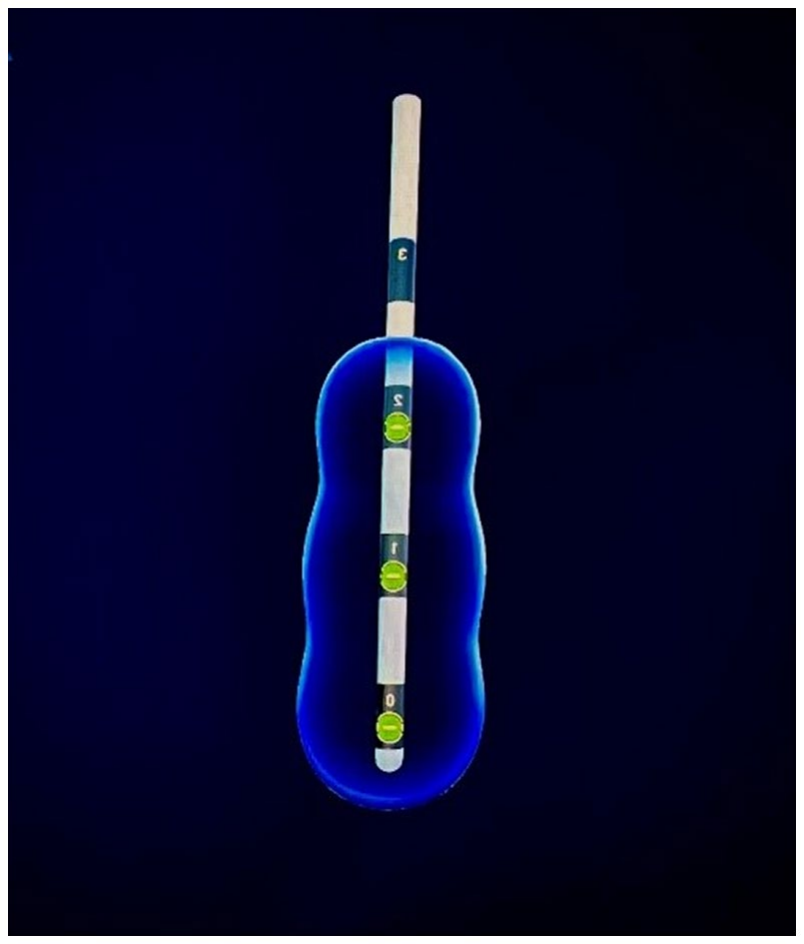
Triple monopolar settings.

Her symptoms remained unchanged at her follow-up approximately 1 month later. Dose optimization continued, now with triple monopolar interrogation using the three most ventral contacts, holding both pulse width (260 μs) and frequency (135 Hz) constant (See Figure 4). Again, no mirth or smile resulted from any setting changes. On the left, mild nausea was noted at 3.2 mA and resolved at 2.5 mA. On the right, mild nausea at 2.3 mA resolved at 2.0 mA. Again, there was no stimulation-induced euphoria, smile, or mirth for any parameter settings. Given that she was now 5 months post-implantation, that a broad electrical field theoretically covered at least the ventral portion of the ALIC, minimal side effects were noted, and the presumption that further interrogation would be unlikely to uncover any mirth response, these settings were held and considered optimized.

At a return visit 5 months later, the patient reported a robust improvement in her quality of life, a “25% reduction in OCD” symptoms, and even more so for her checking behaviors. Some days she experienced “no symptoms” and felt “like myself, minus the OCD.” She also reported experiencing a 4–5 h per day reduction in compulsive behaviors. She also reported the symptoms of depression were absent, and that her mood was “good.” A repeat Y-BOCS had not been performed for this visit. During this visit it was noted that for the left lead, the pulse width had been inadvertently set at 250 μs rather than 260 μs, a finding deemed inconsequential given her marked improvement such that pulse width was continued with this slight asymmetry, still using long pulse width bilaterally. Her device battery life was at 28%, and an elective repair indication notification was present. She was eager to schedule battery replacement surgery given her response. She opted for a rechargeable battery and had the IPG replacement performed at an outside hospital closer to her home where the IPG was changed to a Boston Scientific, device type– Vercise Genus. Final settings were as follows: Left C+, 0−, 1-, 2- @ 2.7 mA, 250 μs, 135 Hz, Right C+, 0-, 1-, 2- @ 2.3 mA, 260 μs, 135 Hz.

#### Follow-up and outcomes

After her IPG replacement, she also transferred her programming care to an outside team closer to her home. Immediately after the replacement, her new provider changed her device settings from our initial optimization, reducing the longer pulse width. The patient reported they had concerns about the battery consumption due to her prior settings. New settings were: bilateral triple monopoles at the three most ventral contacts, 7.5 mA, 136 Hz, and 150 μs. However, one month after her settings had been changed the patient reported a return of depression and a significant worsening of OCD symptoms. This persisted for several months, and she contacted us requesting a DBS programming consultation. She reported her symptoms were now “debilitating,” with OCD compulsions lasting several hours daily, and that she was in bed for hours at a time with thoughts of suicide beginning to return.

We resumed her DBS programming and began transitioning her settings to her previous parameters in an attempt to replicate the previous response. At the time of this writing, we have continued with the use of triple monopoles bilaterally, and over the next 3 months will transition her settings to a longer pulse width, monitoring for side effects. Vecise Neural Navigator 4 software indicates her most recent settings (9.0 mA, 200 us, 136 Hz) will increase her daily charging time from only 20 to 30 min.

Our goal was to gradually transition her back to her previous optimized settings. During the transfer of care to our clinic, we reintroduced the longer pulse-width settings. However, it had previously taken several weeks to adjust some of those settings. While increasing the pulse width, she experienced hot flashes and nausea that persisted beyond 200 us. As a result, we kept her at 200 us. Her overall dosing was lower than what was currently being used, so we adjusted her amperage as it had minimal effect on the nausea at 200 us pulse width. Two weeks later suicidal thoughts had diminished. After her follow-up 2 months later, her depression and OCD symptoms remained unchanged. So, we targeted a longer pulse width again.

During one of her early optimization trials, we noted that nausea would dissipate by lowering the frequency to around 99-105 Hz. We leveraged this and lowered her frequency at the 2nd follow-up visit after returning to our care; this allowed us to achieve a longer pulse width (260us) without side effects or nausea. Communication with the patient over the next 2 months revealed that her depressive symptoms were subsiding with the longer pulse width. She reported that her suicidal ideation had completely disappeared, and she was now out of bed. She described her residual depression as dysphoria arising specifically from her OCD symptoms. However, her OCD symptoms remained unchanged.

#### Timeline


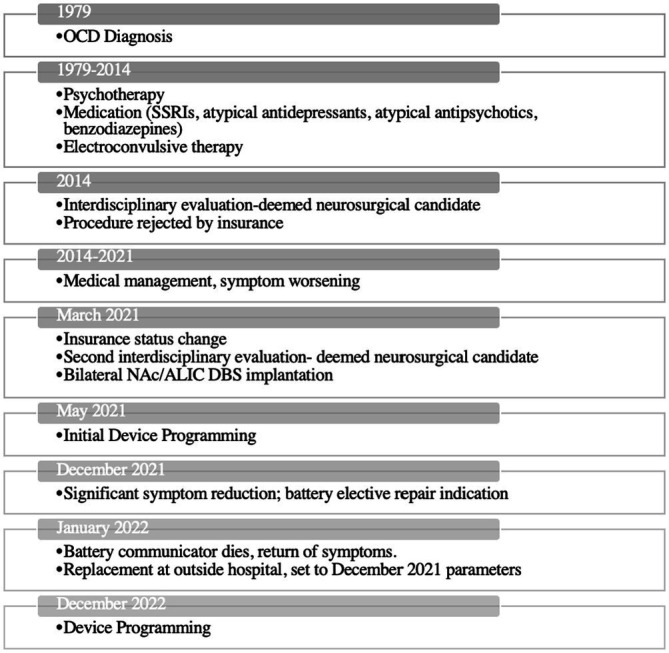


## Discussion

### Target overview

Although the ALIC/NAc is the general region of DBS placement for the treatment of OCD, the precise targets and mechanism of action are still unresolved. Complicating matters is that device interrogation for psychiatric disorders lacks the immediate objective feedback seen in the treatment of motor disorders given the phenomenological nature of psychiatric illnesses. This adds to uncertainty, not only about the region to be targeted but also about whether the correct target has even been stimulated. Despite a precise lead placement, the ALIC with or without the addition of the NAc remains a broad and vague target. Moreover, the induced electrical field within it can vary significantly with only slight variations of lead placement or stimulation parameter change. For example, subtle stimulation changes at the most ventral lead may potentially encroach not only into the NAc but may spread to other unintended areas. Therein, DBS programming must also consider stimulation effects, including any induced acute side effects–in addition to merely creating an electrical field within the ALIC.

One case report noted a non-response for depressive and obsessive symptomatology when solely the NAc was stimulated, yet noted a response after the ventromedial caudate nucleus contacts were activated (16, 17). Furthermore, a study of stimulation versus sham-induced side effects of DBS for OCD noted mood effects with acute stimulation of both the dorsal and the ventral ALIC, as well as the NAc, with worsened mood associated with most ventral lead (nearer NAc), and the middle contact (within ALIC) associated with improved mood (18). Recent literature suggests that in ALIC/NAc lead placement, stimulation may spread to multiple targets such as the associated bed nucleus of stria terminalis (BNST), and “off-target” effects may contribute to response variation (19). This is likely the case for our patient who had an ALIC/NAc lead placement, and whose most ventral contact likely has current spread into the NAc and beyond.

Locating the ideal target or temporally linking an associated stimulation to the target is complicated by the potential for a lag in the emergence of any stimulation effect. Moreover, to minimize surgical time, per-operative exploration settings often sample fewer and more assertive amplitude configurations than in the outpatient setting. This unrefined sampling provides only a rapid, yet rough, overview of theoretically beneficial or problematical settings. Per-op settings are often not congruent to those sampled during an outpatient visit where there is more time allocation for subtle or delayed effects during the interrogation. These post-op settings allow for smaller, incremental stimulation adjustments to note any effects on patient comfort (i.e., dysphoria or panic) or safety (i.e., emerging mania). Or any emergence of a change in affect that may be quite subtle. Problematically, the kinetics of such limbic effects are dependent on the brain target, and the order or intensity of the stimulation. And a previous stimulation may, to some unknown degree, influence the effects of the next confounding the causal link. This could lead to misinterpretation of any noted or unseen per-op and post-op stimulation effects where these effects have not been explored within similar conditions. Such conditions may also include the environmental setting, order in which contact stimulation is trialed, use of unilateral or bilateral leads, configuration settings, rapidity of amplitude changes, etc., all of which may induce significant differences in the observed clinical effects. Moreover, the induced and observed stimulation effects do not neatly overlay an observed clinical effect. Despite these issues, the association of potential efficacy seen in those patients manifesting a stimulation-induced mirth response led to our systematic search for the same. Although high amplitude doses are not the goal to establish efficacy, too low of an amplitude could result in an inadequate electrical field. As such, even with diminished feedback by a mirth response, titrating to a higher amplitude dosing just beneath any noted side effects would at least assure a maximum tolerated dose was utilized.

### Stimulation-induced euphoria, mirth, smile

Controlling the electrical field shape and intensity is important for proper targeting, and for reducing side effects. The transition towards the use of constant current (versus voltage) for newer devices, diminishes any fluctuations in amperage arising from variation in impedance, thus affording a reduced side effect profile (20, 21). However, it has also been shown that stimulation effects such as mirth, smile response, mood change, or even panic might serve as a beacon for optimizing contact selection and current density parameters, and might suggest higher efficacy. The prognostic value of such stimulation effects intraoperatively or during outpatient programming is currently unknown.As occurred with our patient, two-thirds of DBS lead contacts do not induce notable simulation effects on mood, smile, or produce side effects such as other physiological responses, that would have ideally served as potential biomarkers for dose optimization (12, 18). Moreover, stimulation-induced side effects also serve as a natural limiting factor for amplitude settings, i.e., current density.

Additionally, the interpretation of these subtle subjective emotional changes, i.e., stimulation effects, may be further obscured by the direct effects of stimulation producing an affective marker, during DBS programming. For example, previous work has postulated that involuntary facial muscle movement may be disrupted for OCD patients at the level of the basal ganglia and may be independent of mood-related epiphenomena (22). Several cases of unilateral programming inducing a contralateral smile have been reported, which is suspected to arise from the direct stimulation of limbic-motor loops (18, 23). This phenomenon has been hypothesized to be a predictor of OCD response (24). The smile response is often not an isolated motor event. It may spread bilaterally and later become euphorigenic (12). Studies have further aimed to differentiate between the mechanisms of euphoria and “context-dependent mirth” in DBS (24). One such study mapped euphoria to an array of regions, including the STN, NAc, medial temporal cortex, and hypothalamus, with a greater role for the NAc in euphoria than context-dependent mirth (24).

As with the muscles of facial expression involved in smiling, dysregulation occurs in the behavioral circuitry for laughter in OCD patients, and intraoperative stimulation of laughter has been similarly thought to be a predictor of DBS response (22, 24). During programming optimization, this must be interrogated promptly, as habituation of the laughter response occurs after device activation. This laughter may involve a process occurring at the level of NAc and be related to the loss of novelty response (24). This may be distinct from DBS-induced impulse dysregulation or mania which are both thought to possibly arise from ventromedial STN stimulation affecting limbic areas (21). Though the subtle distinction between spontaneous and context-dependent smiling and laughter relate to their respective neural pathways, whether these contacts are the most optimum targets remains unknown.

During the exploration phase for our patient, we noted no such mirth or smile response. The search for such a marker was part of the rationale for the increasing pulse widths, that were beneath the amplitude for any uncomfortable side effects.

### Stimulation-induced autonomic side effects

Stimulation side effects may place natural constraints on contacts or parameter dosing. This was the case for our patient as we continued to expand the amplitude of her parameters settings up to the side effect threshold (for her, feeling “clammy” or “hot”). Such autonomic phenomena are sweating, sensations of heat and cold, and increased heart rate and breathing occurring, possibly due to autonomic fiber activation associated with the hypothalamus, *via* current spread through amygdalofugal pathways (12, 18). Additionally, amygdala projections and hypothalamic and autonomic fibers in the same circuit terminate on the frontal cortex, where they may elicit a panic response (18). Other commonly seen negative effects of programming are sleep disruption or restlessness (5). In addition to anxiogenic effects, acute dysphoria, and depression have been observed with contacts placed within substantia nigra in Parkinson’s Disease (PD) patients (21). Finally, current spread into the anterior hypothalamus or pathways involving the temporal lobe may be responsible for nausea (7, 18). Irrespective of the stimulation effect as a potential marker of efficacy or merely an unpleasant side effect, the extent to which acute side effects and stimulation response serve as a response predictor have not been verified.

### Battery consumption

It is well-documented that DBS for OCD patients typically requires settings with high power consumption. To minimize psychiatric and surgical risks, preserving the battery life is especially critical for patients without a rechargeable IPG. Without a known marker for optimization, preservation of battery charge is an important consideration, but too conservative an approach might lead to underdosing. In earlier studies, the large current density requisite for treating OCD patients led to the need for battery replacement within 6–12 months (10). This was consistent with the energy consumption of our patient. With battery depletion, relapse is imminent. And the time frame required for spontaneous resolution of mood phenomena arising from device termination, as well as recommendations for settings for DBS tapering are currently unknown and present potential future areas of research (5, 25).

### Parameter selection

While numerous parameter combinations may generate the same charge density, minimizing side effect profile and charge depletion has taken precedence historically. Modifying variables such as current, voltage, amplitude, pulse width, and frequency should attempt to allow for a wider DBS therapeutic window. More precisely, it is an attempt to widen the difference between the minimum stimulation, usually amplitude (of current or amperage) required to produce adverse effects, and the amplitude required to produce a beneficial effect (17, 21). Perhaps the most common and simple approach to increasing charge density is increasing the amplitude of the voltage or the current.

Early seminal trials continue to guide parameter selections, such as the monopolar survey commonly using 130 Hz, 90–210 μs (10, 25). These parameters have natural ceilings for charge density for brain tissue at 30 μC/cm^2^ (25, 26). The use of constant current streamlines programming due to reduced variation in impedance. For example, impedance may be increased at the brain tissue: electrode interface due to increased electrode and IPG encapsulation, which alters the electric field and increases resistance (20, 27). However, there is still a lack of data on parameter variation and anatomical impact (19).

Pioneering work on DBS for essential tremor provided a broad range of frequency parameters (28). At present, frequency typically ranges between 100 and 145 Hz, with some programming work showing 130 Hz as an ideal trade-off between power consumption and clinical efficiency (12, 21). A cross-over study on DBS for treatment-resistant depression found a response advantage with no difference in side effect profile for 130 Hz vs. 20 Hz (29). Previous studies have noted that with the commonly used pulse width of 130 μs, voltages over 5.5 V tend to increase the side effect profile (30). For our patient, we continued with the fairly standard use of 130 Hz. We then began systematically increasing the amplitude of amperage across an array of pulse widths, including longer ones.

However, pulse width selection is highly variable in many studies, with clinical improvement using both short and long pulse widths, including reports of 450 μs in dystonia (17). Additionally, in a study evaluating 26 parameters in DBS for PD, the voltage was the most important factor, and more specifically, maximizing voltage amplitude while minimizing pulse width afforded the most energy-equivalent symptom reduction (31). Most early studies utilized pulse widths ranging between 90 and 210 μs (25). For our patient, we selected a long pulse width as it had been well tolerated during the optimization phase and seemed to allow for an adequately broad therapeutic window. Despite our attempts to elicit stimulation effects (such as mirth or smile), such effects were never elicited. We continued settings with the well-tolerated longer pulse width in the event there was the potential for more current spread across the ALIC and to avoid potentially suboptimal dosing. Three months later, these settings coincided with a notably robust response.

## Conclusion

Our systematic optimization approach resulted in a positive response from the patient. Using long pulse widths, we increased settings to the highest tolerable threshold before side effects were noted. Although this helped assure we had increased the settings as high as was tolerable to avoid under-dosing, higher doses do not necessarily equate with efficacy. Fortunately, the high battery consumption issues plaguing such DBS cases have been mitigated with rechargeable batteries. However, in this single case, the contribution of efficacy arising from the effects of micro lesioning during lead implantation, or the placebo effect is unknown. Yet, her severe symptomatology remained durable past 6 months of optimization, the durability beyond this has not yet been assessed. It is also unclear whether reintroducing her previously optimized settings will return her to a remitted state. For our patient, leveraging the higher pulse width over amplitude, coincided with response, and seems a viable strategy. We offer this as our approach to this particular case. However, with such vast parameter options, and high anatomical variability between individuals and their lead placement, individualized optimization may take a different parameter formulation for others– depending on side effects and response.

## Data availability statement

The original contributions presented in the study are included in the article/supplementary material, further inquiries can be directed to the corresponding author.

## Ethics statement

Written informed consent was obtained from the participant/patient(s) for the publication of this case report.

## Author contributions

BC and EB wrote the first draft of the manuscript. BC conceptualized the discussion section. KP provided clinical resources for the case report. LK helped write and edit manuscript drafts. All authors contributed to the article and approved the submitted version.

## Conflict of interest

The authors declare that the research was conducted in the absence of any commercial or financial relationships that could be construed as a potential conflict of interest.

## Publisher’s note

All claims expressed in this article are solely those of the authors and do not necessarily represent those of their affiliated organizations, or those of the publisher, the editors and the reviewers. Any product that may be evaluated in this article, or claim that may be made by its manufacturer, is not guaranteed or endorsed by the publisher.
